# 

**Published:** 1891-05-30

**Authors:** 


### NOTICE TO CORRESPONDENTS.

All MS., Letters, Books for Review, and other matters intended for the Editor should be addressed THE EDITOR, THE LODGE, PORCHESTER SQUARE, LONDON, W.

The Editor cannot undertake to return rejected M.S., even when accompanied by stamped directed envelope.

**Notice to Advertisers and Subscribers.**

All Advertisements, Orders for Copies of THE HOSPITAL, and Business Communications should be addressed to The Publisher, *not to the Editor*, THE HOSPITAL, 140, Strand, W.C.

The Cost of Subscription, post free, is as follows:—Per week, 1½d.; per quarter, 1s. 8d.; per half-year, 3s. 3d.; per annum, 6s. 6d. Foreign:—Per annum, 8s. 8d.; payable, in all cases, in advance.

**NOTICE.—The Index for Vol. IX., (ending March, 1891) is on sale at the Office of this Journal, price 2½d., post free.**

### GENERAL CHARITIES.

[This Column is devoted to an account of work of charitable institutions other than Medical Charities. The Editor will be glad to receive reports or news of work at 140, Strand. *Philanthropy* should be plainly written on the envelope.]

**THE CRIPPLED BOYS' HOME**, Kensington, has received a donation of £50 from the Goldsmiths' Company, and £10 10s. from the Skinners' Company.

We do not, like Mr. Carnegie, argue for the advantages of poverty; poverty in moderation is one thing, hunger, thirst—unsatisfied—another. We do not ask for charity to be "organised" to such an extent that it becomes heartless, hard and fast, relentless, with an ever growing suspicion which ends in believing everybody to be a mere plausible humbug. We congratulate ourselves in England and say "how charitable we are," and if the number of so-called charitable institutions is a guide, then we certainly are. But there is something wrong somewhere when new schemes are enabled to jump into existence every day, while those who have borne the heat and burden of the day are left to ask for funds in vain.

The forty-fourth anniversary festival in aid of the funds of the **ASYLUM FOR IDIOTS**, Earlswood, Redhill, was held last week, under the presidency of Mr. J. Blundell Maple, when subscriptions amounting to £4,000 were received. Everybody knows how inadequate the relief for people of imbecile mind is in this country, and everybody ought to know how admirably the Earlswood Asylum is managed, and what efforts are made by kindness and science to ameliorate the condition of the 600 patients. There is something so pathetic in the very inability of these sufferers to help themselves, and now about 150 people are seeking admission. Would that the public would enable such institutions as Earlswood to spread their endeavours, instead of giving indiscriminately to the first mushroom undertaking which takes their fancy. It is interesting to know that the programme of the music used at the festival was printed by the inmates of the asylum.

Of all cities in the world London is in the proud position of possessing the greatest number of charitable institutions, and of giving shelter to the greatest amount of poverty. There is much food for reflection in these incontrovertible facts. However much our charities are increased in number, however much the amount of money given to them is added to year by year, there still remains with us an increasing, rather than decreasing, mass of the direst poverty, combined, as must inevitably be the case, with the most abject misery. It is equally clear that this should not be, and that there must be something radically wrong with our charitable system. There is a suspicion, which is fast growing in the minds of many into a conviction, that we are rapidly creating a huge pauper caste; that we are sapping the very first principles which keep a nation healthy and vigorous, and are destroying all feelings of self-reliance, planting in their stead an ever-growing feeling of dependence. The multiplication of charities, the only *raison d'etre* of whose existence is that they may reflect some glory on their self-seeking founders in the minds of a thoughtless public, every ready to fall a prey to some new sensation, without staying to question the need of such charity, inevitably creates new wants. If we are to stamp out poverty, especially the growing class of professional paupers, we must inculcate, and not destroy, the practice of self-help, put an end to the present disorganisation of our charitable system, and the consequent overlapping which allows the creation of mushroom institutions at the expense of solid societies which have proved their worth and the need of their existence. As things are, experienced workers among the truly poor are left to struggle, with scant encouragement and help, against the unwisely distributed alms of fatuitous people, who judge of the needs of a case by the amount of emotion aroused in their breasts by a well-spiced and sensational appeal. It is an appalling and discreditable fact that nearly half the working population of this country over the age of 60 has to go to the parish at some time or another for relief. No doubt this is due in no small measure to the absence of almost all principles of thrift amongst the labouring population, but the evil is as undoubtedly aggravated by a system which tends to stamp out independence. A system of national insurance, which would help people to look rather to their own exertions for provision against a rainy day than to extraneous quarters, is probably a long way from consummation; but if more of our charities were based on the mutual system, the difficulties which stand in the way of a scheme which most people think can only be established on a voluntary basis will rapidly disappear. It is a truism rather than a paradox, that charity often creates more distress in the long run than it relieves at the moment. It has been the fashion with some of the present generation to hug themselves in the happy but pharisaical belief that their forefathers, as compared with them, were indifferent to distress; but if former generations can be twitted with indifference, we are in danger of being accused by posterity of blind thoughtlessness. For those who are ready to make some sacrifice of unselfishness in giving, it is generally enough to be informed that the institution they are asked to support is *benevolent*; from many it would be hopeless to ask them to take the trouble to inquire if the undertaking is *beneficent*. Many people are too apt to think that as soon as they have given their money all responsibility for it is at an end, whereas the responsibility really begins with the gift.

### SCRAPS AND GLEANINGS.

THERE is an epidemic of cholera at Manipore.

THE fruit crop around Taunton has been almost ruined by the recent frost.

A BAZAAR has been held in Southampton in aid of the funds of the Eyes and Ear Hospital.

DR. MEADE has been elected Doctor to Templmartin Dispensary in the place of the late Dr. Wall.

SIX cases of cholera have occurred among some Indian pilgrims at the Lazaretto, on Kamaran Island.

THE collections on Hospital Sunday in Carlisle amounted to £362 19s. 8d. and the Hospital Saturday Fund to £22 14s.

THE Goldsmiths' Company have voted £50, and the Skinners' Company £10 10s. to the funds of the Crippled Boys' Home, Kensington.

IN consequence of the epidemic of influenza, the whole of the Law Courts are undergoing a thorough course of fumigation and ventilation.

THE Quarterly General Court of the Governors of the Middlesex Hospital was held on Thursday last, to transact the usual business of the charity.

THE annual meeting of the Funeral Reform Association will be held at Grosvenor House on June 2nd under the presidency of the Duke of Westminster.

OUT of the 102 inmates of the Wirrall Workhouse, Cheshire, close upon 100 have been attacked by influenza, and nine deaths have occurred during the past fortnight.

THE influenza epidemic is rapidly abating in the Yorkshire towns, where it first manifested itself, and the medical men have now scarcely any influenza cases on their books.

OWING to want of funds the Zenana Medical College has lately been obliged to limit its usefulness and decline the services of several suitable candidates for medical mission work.

THE annual bazaar and sale of work in aid of the Dublin Deaf and Dumb Association, was held in Dublin on the 13th and 14th of May. The attendance was not so good as in former years.

DR. REDARD, of Geneva, now substitutes chloride of ethyl for chloride of methyl, in producing local anæsthesia by refrigeration, as chloride of methyl requires an expensive apparatus to utilise it.

THE death rate in Sheffield for the week ending May 16th shows a considerable decrease, being at the rate of 34 per 1,000 per annum, as against 59 the previous week, and 70 the week before that.

THE foundation stone of the Rose-Innes Cottage Hospital, Marnoch—an institution which is to be built by means of an endowment left by the late Miss Rose-Innes, of Netherdale—was laid on the 18th inst.

THE Committee of the Metropolitan Benefit Societies' Asylum have just received a cheque for £500 from Mr. John Saul, of Tottenham, being a third of the £1,500 he has promised to contribute to the funds.

ON Saturday, June 5th, a grand concert will be given at Grosvenor House, by permission of the Duke of Westminster, in aid of the "Countess of Dufferin's Fund" for supplying medical aid to the women of India.

THE returns of the Medical Officer for Health in Manchester, show that during the week ending May 9th the births were fewer by 22, and the deaths more by 135, than the average weekly numbers of the previous six weeks.

A SERIOUS attack of rabies has occurred at Wincanton, Somersetshire On May 8th a rabid dog from Sherborne, Dorset, appeared at that place, and was not destroyed until it had bitten a large number of other dogs.

"BEAUTY as a Means of Health" was the subject of a lecture recently delivered to a working girls club in New York by Dr. Louise Fiske Bryson who affirms that systematic efforts to be beautiful will secure a fair degree of health.

MR. BLUNDELL MAPLE, M.P., presided at the annual festival of the Earlswood Asylum, held at the Hotel Metropole on the 20th inst. During the evening donations amounting to £3,956 were announced, and the Chairman at once raised it to £4,000.

DR. ALICE KER, known to many in Manchester through her valuable lectures at the School of Domestic Economy, has been appointed hon. surgeon to the Birkenhead Lying-in Hospital, in addition to her former appointment as medical officer to the Wirral Children's Hospital.

THE next meeting of the Odontological Society of Great Britain will be held on Monday, June 1st, at eight p.m., when Mr. F. H. Balkwill, L.D.S.Eng., will read a short paper of "Notes on Some Morphological Dental Irregularities in some of the Skulls in the Museum of the Royal College of Surgeons of England."

THE medical career is now open to women in all parts of Russia, and special privileges are granted to women doctors in all institutions, schools hospitals, and charitable foundations for women. They are exempted from being compelled to give their services in criminal cases as their brother physicians are, but must, however, always wear a badge as a sign of their profession.

AT a meeting of the Board of Management of the Salford Royal Hospital, it was announced that a cheque for £2,000 had been received by the Treasurer from the executors of the late Mrs. Margaret Platt, of Stalybridge, to be devoted to the general purposes of the institution. The Treasurer reported that the bank account was over-drawn to the amount of £3,869 13s. 5d., so that this legacy will considerably reduce the balance owing to the bank. The income of the Hospital is more than £4,000 under the expenditure.

THE fourth annual report of the Glasgow Hospital for Diseases peculiar to Women, states that the position of the hospital is greatly improved through having removed to the commodious and well-appointed premises at 29, Elmbank-crescent. During the year 1890 106 operations, requiring treatment in the wards, have been carried through with only one death, and that in a woman who was moribund on admission. Three thousand, four hundred and thirty-eight consultations, combined in each instance with treatment and appliances suitable to the case, have been given. The income from all sources amounted to £511 18s. 1½d., and the expenditure to £443 12s. 2½d.

